# Neuronal imaging with ultrahigh dynamic range multiphoton microscopy

**DOI:** 10.1038/s41598-017-06065-7

**Published:** 2017-07-19

**Authors:** Ruohui Yang, Timothy D. Weber, Ellen D. Witkowski, Ian G. Davison, Jerome Mertz

**Affiliations:** 1 Boston University Department of Biomedical Engineering, 44 Cummington Mall, Boston, MA 02215 USA; 2 Boston University Department of Biology, 5 Cummington Mall, Boston, MA 02215 USA; 3 Boston University Photonics Center, 8 St. Mary’s St., Boston, MA 02215 USA

## Abstract

Multiphoton microscopes are hampered by limited dynamic range, preventing weak sample features from being detected in the presence of strong features, or preventing the capture of unpredictable bursts in sample strength. We present a digital electronic add-on technique that vastly improves the dynamic range of a multiphoton microscope while limiting potential photodamage. The add-on provides real-time negative feedback to regulate the laser power delivered to the sample, and a log representation of the sample strength to accommodate ultrahigh dynamic range without loss of information. No microscope hardware modifications are required, making the technique readily compatible with commercial instruments. Benefits are shown in both structural and *in-vivo* functional mouse brain imaging applications.

## Introduction

**Figure 1:**
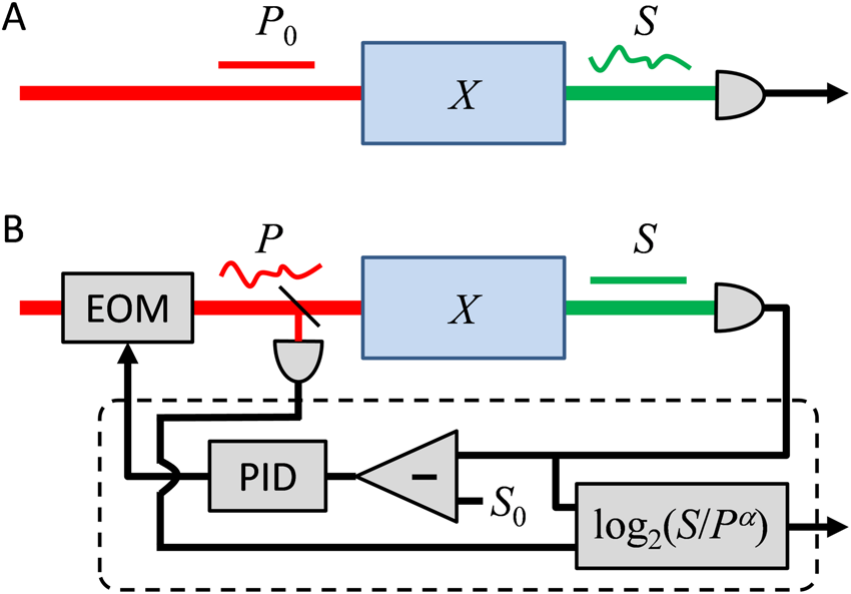
Schematic of principle. Layout for (**A**) conventional, and (**B**) active-illumination-assisted multiphoton microscopy (PID = proportional-integral-derivative feedback).

Multiphoton microscopy has become the most common and effective method for high-resolution functional brain imaging because of its remarkable depth penetration in thick tissue^1^. In standard configurations, such microscopy involves scanning a femtosecond laser focus in 3D throughout a sample. The laser power is fixed during the scan and image information is contained in the time dependence of the detected fluorescence signal (Fig. 1A). Several problems can occur with this technique. First, in common cases where the sample contains extreme variations in brightness, for example between large somas and much finer dendritic processes, it is often impossible to capture the full range of signals without either saturating the detector when scanning over bright regions, or losing signal when scanning over dim regions. Second, when imaging time-varying signals from functional reporters such as GCaMP^2^, large brightness variations occur that cannot be predicted in advance, forcing the user to operate with low illumination power to minimize the possibility of detector saturation, thus compromising SNR. Third, when performing volumetric scans through an extended range of depths, a single laser power becomes either too weak at large depths or too strong at shallow depths.

A simple solution to all these problems involves actively regulating the laser power pixel by pixel using negative feedback electronics^3, 4^. However, to date, implementations of this solution have been based on custom analog electronics that have been difficult to build and calibrate, leading to image reconstruction that was unreliable. We present here a modified technique based on simultaneous detection of the signal and illumination powers that is user-friendly, calibration-free and can be assembled from readily available off-the-shelf components. Our solution is a self-contained unit that can be attached to any multiphoton microscope, commercial or otherwise, with no hardware modifications whatsoever. Our only assumptions are that the microscope is based on standard (i.e. non-resonant) galvanometric scanning and that it is equipped with a method to rapidly control laser power (for example, most commercial vendors provide power control with an electro-optic modulator (EOM)), and a simple photodiode to probe the laser power. Because our unit is based on digital field-programmable gate array (FPGA) electronics and dual detection of signal and illumination powers, its operational parameters are well-defined and image reconstruction is robust and accurate.

## Technique

Our strategy, which we refer to as active illumination, is illustrated in Fig. 1B. We define the fluorescent sample strength *X* to be a variable that includes all factors contributing to the local fluorescent emissivity, including concentration, cross-section, quantum yield, etc., such that the detected multi-photon excited fluorescence is *S* = *XP* ^*α*^, where *α* is the excitation order (2 for two-photon microscopy). Negative feedback is used to hold the detected fluorescence to a constant *S*_0_ by controlling the input illumination power, up to a user-defined maximum power *P*_*max*_. When sufficient power is available to hold *S*_0_, the system is in feedback active mode. When more than *P*_*max*_ would be needed to reach *S*_0_, the power is automatically set to *P*_*max*_ and the system switches to power-limited mode. In either case, the desired quantity of interest is the sample strength *X* = *S*/*P* ^*α*^. This is evaluated directly in the electronic unit and supplied as an output. In other words, the user need not be concerned whether the system is in feedback-active or power-limited mode – the output remains the desired *X* regardless. Moreover, even if the feedback fails to some degree and neither *S* nor *P* attains its targeted value, the ratio *X* remains correct, which is all the user cares about.

While the strategy outlined above is useful for limiting the exposure of the sample to unnecessary illumination, and thus limiting the possibility of photobleaching or phototoxicity (a similar strategy involves on/off illumination control^5–7^), it does nothing to improve the dynamic range of the acquired images. For this, an additional feature to our strategy is required. The problem is made clear with an example. Let us consider the standard case where microscope signal acquisition is performed with a 12-bit A/D converter. In this case, even if the optical detector (typically a PMT) is noiseless, the best dynamic range one can hope to achieve is 4096:1. And even if one were to switch to a converter with larger bit depth, one would then run into dynamic range limitations imposed by the detector itself, which for a PMT is typically 10^4^–10^5^. These ranges are often short of the brightness ranges occasioned in fluorescently labeled brain tissue, sometimes by orders of magnitude.

**Figure 2:**
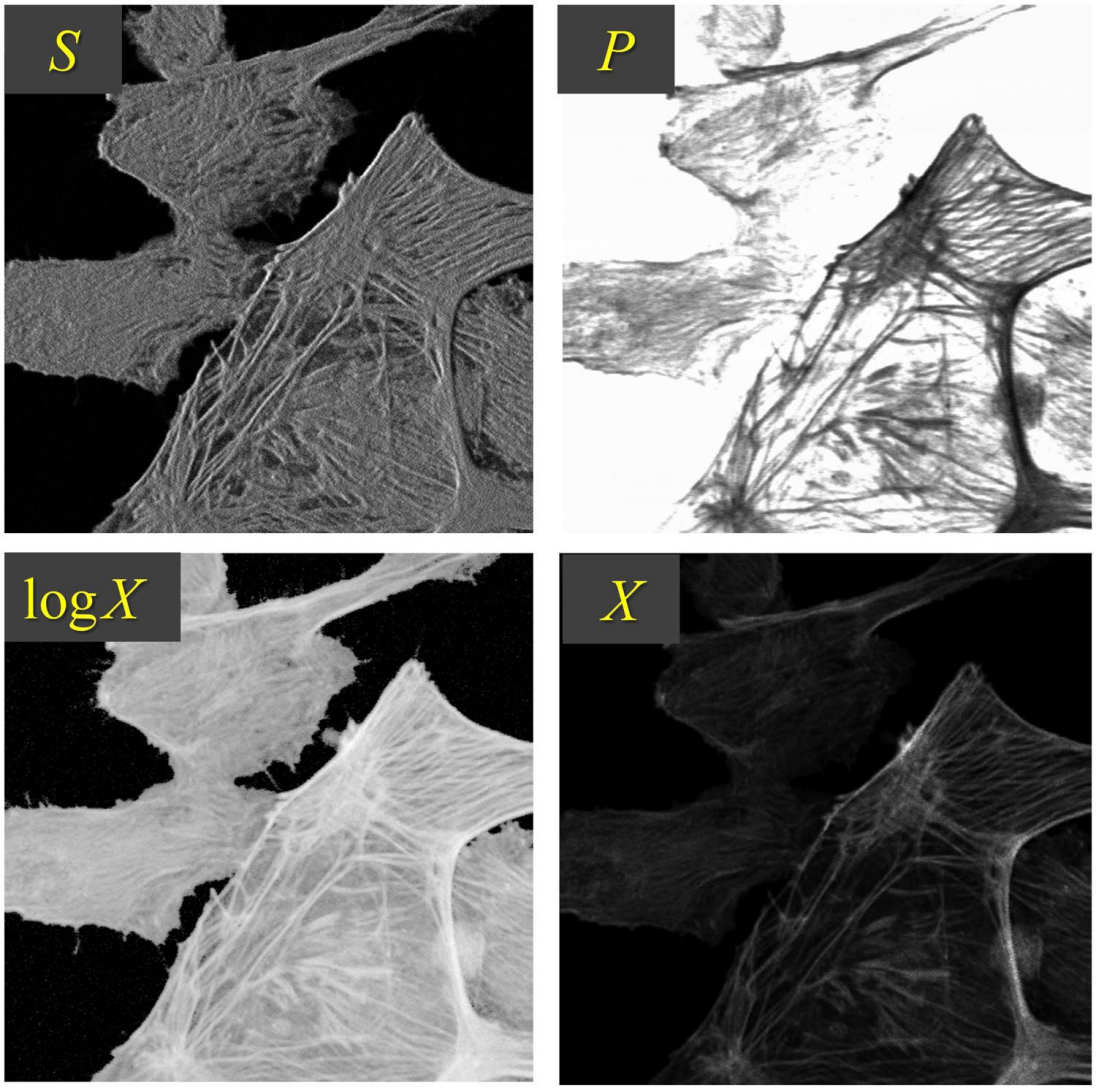
Demonstration of technique. *S* is the raw PMT data obtained from BPAE cells (FluoCells Prepared Slide 2, Molecular Probes) while active illumination is on (normally this signal would be sent to the microscope acquisition electronics). *P* is the illumination power measured by the photodiode. ${\mathrm{log}}_{2}X$ is the resultant log-encoded sample strength sent to the microscope acquisition electronics. *X* is the linear-encoded sample strength calculated post-hoc (see below). Note the bright edges apparent in *S* are caused by overshoot in the feedback at 4 *μ*s pixel time (see Supplementary Information for details). These are canceled after computation of ${\mathrm{log}}_{2}X$ (or *X*).

**Figure 3:**
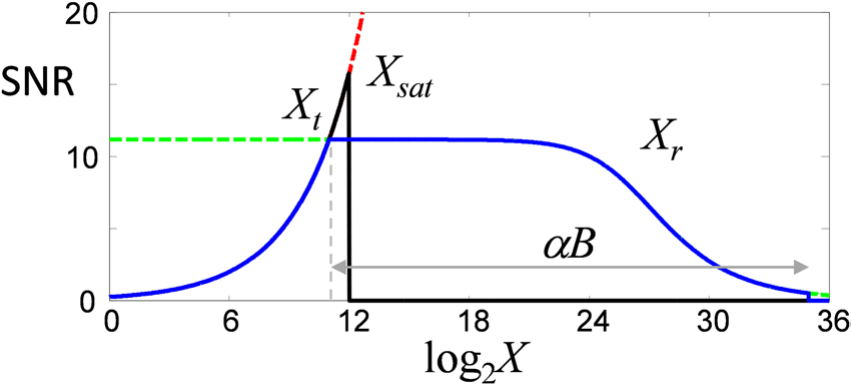
Analysis of signal to noise ratio (SNR) as a function of sample strength *X*. Black trace is SNR for conventional two-photon microscopy (*α* = 2), limited to sample strength *X*_*sat*_ at which point the detector saturates, for acquisition bit depth *B* = 12. Red and green dashed traces are SNR for power-limited and feedback-active modes respectively – transition between the two modes occurs at *X*_*t*_. Net AI SNR (blue trace) is shot-noise limited up to sample strength *X*_*r*_, at which point it begins to roll off when the laser power *P* falls below detector noise or becomes undersampled. Signal set point *S*_0_ is set here to half the detector saturation level. The maximum allowed laser power *P*_*max*_ is set to *P*_0_. *S*_*sat*_ is taken to be 250 (see Supplementary Information). Note that conventional microscopy leads to higher SNR only for a small range of sample strengths. This range can be reduced by increasing *S*_0_.

When examining the problem more closely, one realizes one could do much better, even with a standard 12-bit converter. The problem is that the converter is not efficiently utilized. As the optical signal increases, so too does the shot noise associated with the signal, meaning that the fine sampling provided by the 12-bit converter becomes wasted at high signal levels where all it does is oversample noise. A much better strategy is to redistribute the sampling so that fine sampling is provided at low signal levels where it is needed, while coarser sampling is relegated to larger signal levels. Such a redistribution of sampling can be achieved by applying a nonlinear (more precisely, sub-linear) operation to the signal before it is sampled by the linear A/D converter. The operation we choose for our system is the logarithm operator (see Fig. 2). To be clear, we apply the logarithm operator to the sample strength *X*. But in turn, this is derived from the logarithms of the fluorescence signal *S* and the laser power *P* by the simple relation ${\mathrm{log}}_{2}X={\mathrm{log}}_{2}S-\alpha {\mathrm{log}}_{2}P$. We emphasize that such a redistribution of gray levels entails no additional loss of information, since, up to sample strengths much larger than *X*_*sat*_, loss of information remains dominated by uncertainties due to shot noise rather than inaccuracies due to sampling (see Fig. 3).

A theoretical evaluation of the dynamic range provided by our system is provided in Supplementary Information, and illustrated in Fig. 3 for the case when *S*_0_ is set to half the detector saturation level. The resulting maximum gain in dynamic range compared to standard multi-photon microscopy is given by ×2^(*αB*−1)^, where *B* is the bit depth of the microscope acquisition electronics. For two-photon microscopy with typical bit depth of 12, this corresponds to a maximum gain in dynamic range of almost 10^7^. For three-photon microscopy^8^, the increase would be greater than 10^10^.

## Results

**Figure 4:**
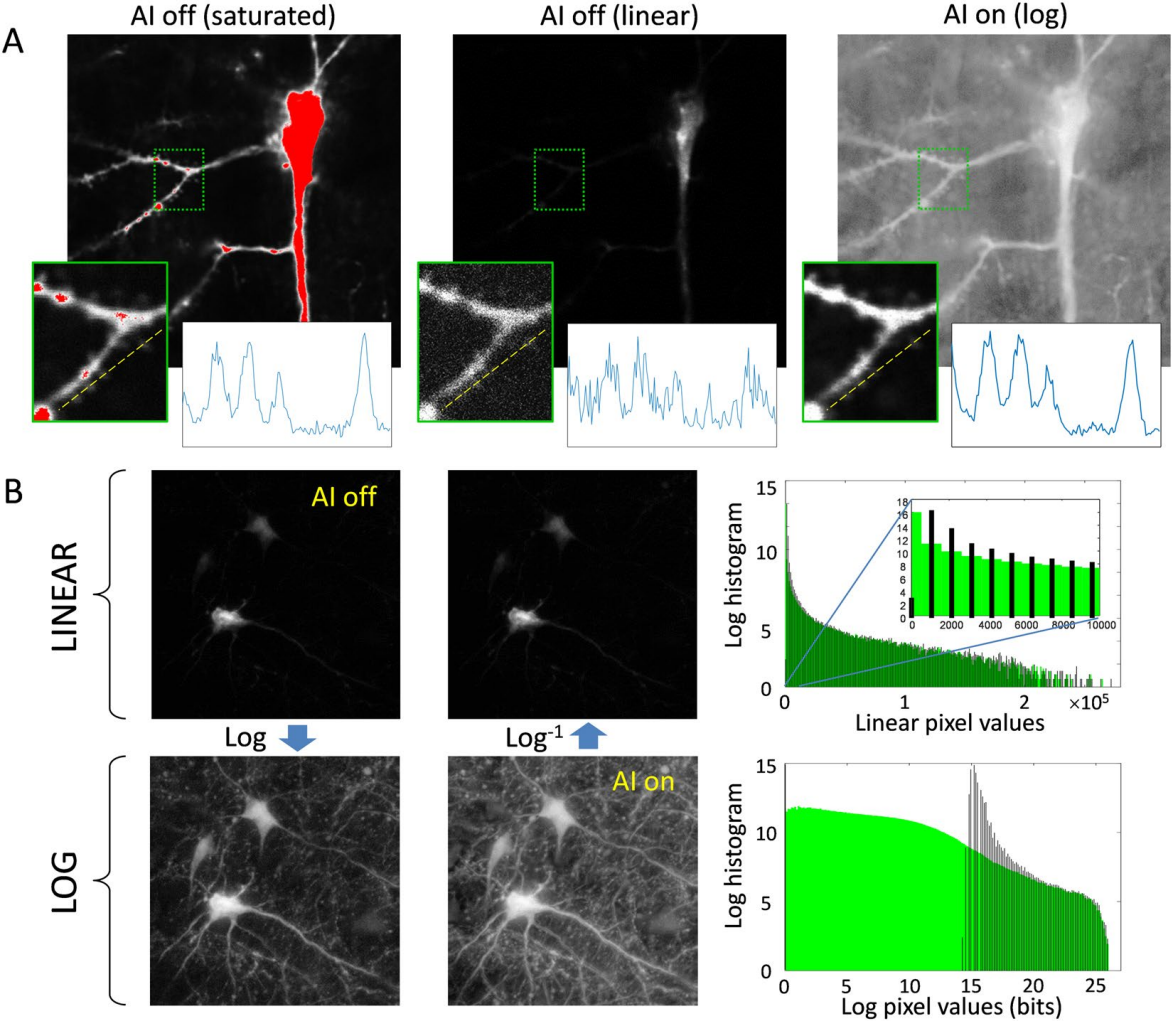
Demonstration of high dynamic range mouse brain imaging. (**A**) YFP-labeled pyramidal neuron in neocortical slice (single frame from a 26-frame 75 *μ*m z-stack, 3.2 *μ*s pixel time – Supplementary Video 1) acquired with high (left) and low (center) laser power, and active illumination (right). High power reveals neural processes but causes detector saturation (red), whereas low power (just below saturation) produces a mostly dim image. AI (log scale) provides much higher dynamic range while never saturating. Re-scaled insets are shown for comparison, following post-hoc linearization of the AI-on inset, along with plot profiles of four spines (along dashed yellow line). High power and AI images exhibit similar SNR (slight differences are attributable to sample drift between acquisitions), whereas spines in the sub-saturation power image are barely discernable. (**B**) YFP-labeled neurons (maximum intensity projection of a 23-frame 88 *μ*m z-stack, 3.2 *μ*s pixel time – Supplementary Video 2), acquired conventionally (AI off, linear, just below saturation) and with active-illumination (AI on, log). For comparison, images are also shown in log and linear representations, respectively. Histograms of the linear images appear almost identical, whereas histograms of the log images reveal that in fact AI (green) provides here a 26-bit dynamic range compared to the 12-bit dynamic range provided by conventional acquisition (black).

We demonstrate some benefits of active illumination when applied to neuronal imaging with commonly used genetic indicators. During structural imaging of neuronal anatomy, different cellular compartments often lead to a range of signal intensities greatly exceeding what can be captured by current multiphoton microscopes. For example, dendritic spines are a widely used readout of neural plasticity^9^, but these small and dim structures are often difficult to resolve without saturating signals from larger adjacent compartments such as parent dendrites or the cell body. This problem is remedied by active illumination, as illustrated in Fig. 4, greatly improving the SNR of spine imaging while preventing clipping of signals from larger dendritic branches. Indeed, the sample strengths in Fig. 4B span a range $\sim \,{10}^{8}\mathrm{:1}$. The display of such a large dynamic range is facilitated with our log representation, and a conventional linear representation can be readily recovered post-acquisition by applying the antilog operator to *X*. Importantly, the overlay of linear histograms in Fig. 4 confirms that the fluorescence levels acquired with and without AI are reliably consistent over the full span of brightness ranges. That is, AI allows both bright and dim cellular compartments in mouse brain tissue to be simultaneously and accurately quantified without the need for repeated exposures.

**Figure 5:**
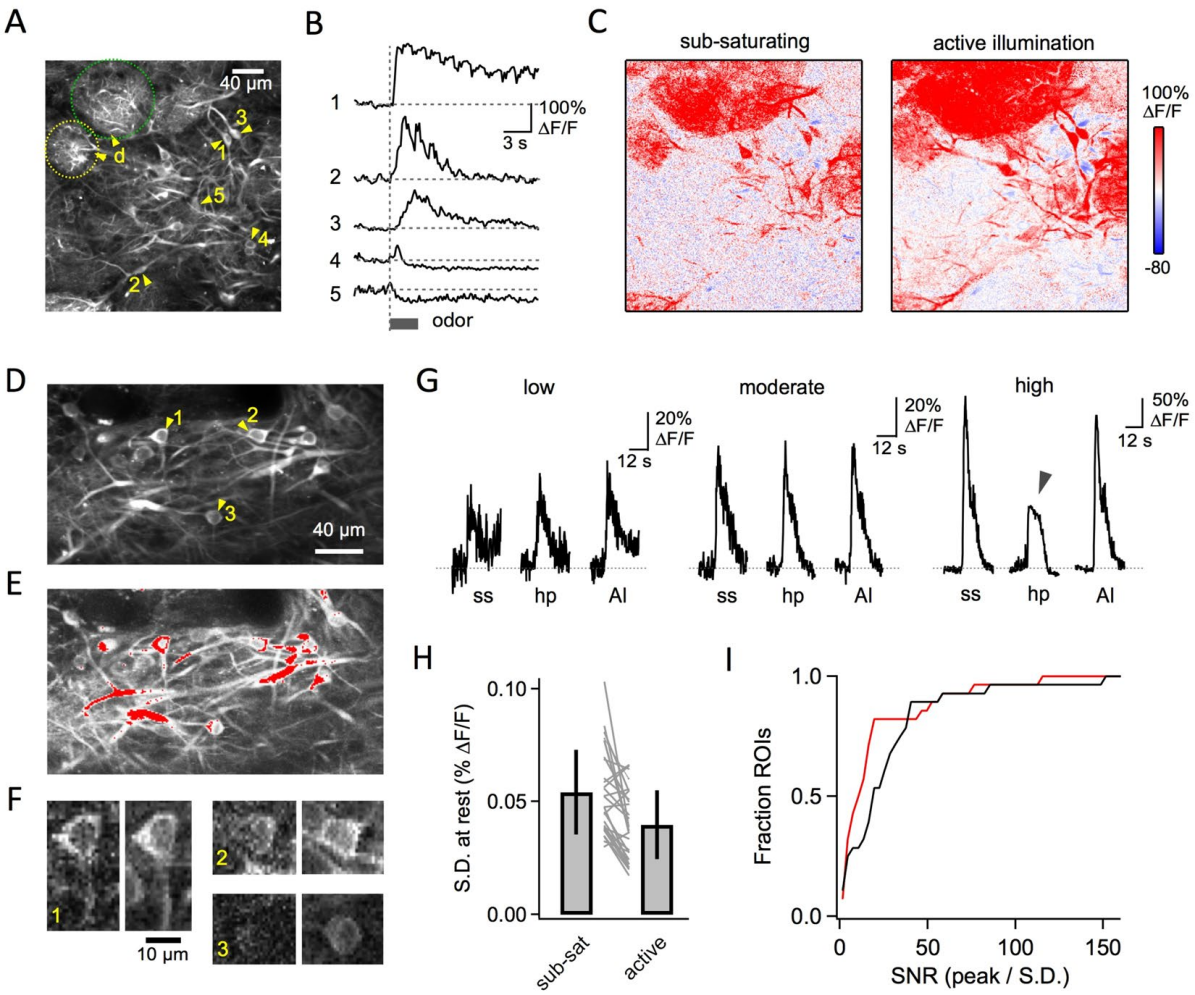
Improved functional imaging of neural activity with active illumination. (**A**) Mitral/tufted cells and dendrites in the olfactory bulb of Thy1-GCaMP3 mice (3.2 *μ*s pixel time – Supplementary Videos 3–5). (**B**) Sensory responses to odors for selected ROIs (arrowheads) exhibit both increases and decreases in activity spanning a wide intensity range. (**C**) Activity maps showing average Δ*F*/*F*_0_ during the 5 sec period after odor stimulation: when the laser power is limited to prevent saturation, activity maps primarily reflect only the strongest-responding structures (left); when the laser power is increased with AI, the improved SNR enables the detection of smaller changes in soma and dendrites, providing a more detailed identification of activity across the neural population (right). (**D,E**) Increasing laser power alone leads to detector saturation in many areas, where red identifies pixels clipped at any time during the image sequence (3.2 *μ*s pixel time – Supplementary Videos 6–8). (**F**) Increased image quality with AI, shown for three example neurons in single imaging frames prior to odor stimulation. (**G**) Comparison of sensory responses in 3 different neurons with and without AI, showing cells with low, moderate, and high activity levels. AI prevents clipping at higher illumination intensity (right, arrowhead). (**H**) On average, AI gave a ~30% reduction in resting noise levels of individual ROIs. (**I**) Cumulative SNR histograms showing rightward shift toward higher SNR with AI (black) compared to without AI (red). SNRs were calculated for each ROI as peak Δ*F*/*F*_0_ during the odor response divided by the standard deviation (SD) of the resting activity.

Active illumination also offers substantial benefits for imaging neural activity with genetically encoded Ca^2+^ indicators. Activity levels often vary dramatically across different neurons within a circuit^10^, posing challenges for capturing both large fluorescence increases within the most highly active cells without sacrificing signals from weakly active neurons that still encode meaningful information. This problem is compounded by the increased dynamic range of the latest generation of Ca^2+^ indicators such as GCaMP6^2^. To illustrate this situation, we imaged sensory responses to odors in the olfactory bulb of Thy1-GCaMP3 mice^11^, where neurons exhibit both increases and decreases in neural activity with diverse amplitudes and temporal dynamics (Fig. 5A,B). Active illumination enabled the use of sufficient laser power to reveal weakly activated cells while at the same time preventing the clipping of more strongly responsive cells. Active illumination also provided improved resolution of Ca^2+^ changes in smaller dendritic compartments (Fig. 5C), which play important roles in integrating input from other neurons^12^ and can even act as independent input-output sites^13, 14^. Overall, active illumination decreased the noise levels in resting signals from individual cells, while providing a corresponding increase in the SNR of sensory responses (Fig. 5D–I), illustrating an increase in the amount of information about circuit dynamics that can be extracted with Ca^2+^ indicators.

## Discussion

The pixel sampling rates achieved by our system are as high as 1 MHz (see Supplementary Information and Supplementary Video 9, acquired at 1.2 *μ*s pixel time), limited by the bandwidth of our EOM. Such rates are easily on par with what is generally employed with non-resonant-galvanometer-based microscopes. Faster rates still, such as video rate, could be accommodated by our FPGA electronics and are attainable in principle, but would require an EOM of bandwidth approaching 10 MHz and a detector of shorter response latency than our PMT.

One might wonder how it is possible to improve dynamic range beyond the intrinsic range of the detector acquisition electronics. The key here is that two detectors are used, not just one. Moreover, the signal of interest, namely the sample strength *X*, depends on the simultaneous measurements provided by both of these detectors, not just one. In the case of multiphoton microscopy the dependence on one of these measurements is nonlinear, further increasing the sensitivity to this measurement.

We did not attain the full potential gain in dynamic range predicted by theory (see Fig. 3) for a variety of reasons. First, we gave ourselves some wiggle room (*X*_*t*_ < *X*_*sat*_) to allow for the possibility of errors in the feedback, such as feedback overshoot. Second, it was necessary to introduce a constant offset to log_2_ *X* because our microscope acquisition electronics did not allow negative input levels (see Materials and Methods). Third, a roll-off in SNR occurred at *X*_*r*_, caused by our inability to precisely control and detect the illumination power *P* at levels when it is very small (owing in part to digitization inaccuracies, noise in the EOM control and photodiode electronics, and a weak quadratic dependence of the EOM response at that level). Nevertheless, the gains in dynamic range we achieved are substantial. For example, a gain of ×2^14^ is illustrated in Fig. 4B, above and beyond the range of 2^12^ provided by standard microscope acquisition electronics.

Compared to multi-exposure^15^, multi-signal-attenuation^16^, or post-detection^17^ strategies for achieving high dynamic range, our technique provides the added benefits of limiting photobleaching or phototoxicity in the sample (*P*_*max*_ is set to no higher than the illumination power *P*_0_ normally used in standard imaging – see Supplementary Information), and reducing potential of damage to the detector itself caused by sudden signal transients (*S* is prevented from being larger than *S*_0_). It should be noted that active illumination can also be applied to single photon scanning microscopy (e.g. confocal), however with reduced benefits to dynamic range and photodamage limitation.

The purpose of our active illumination add-on is to enable scanning microscopes, particularly multiphoton microscopes, to see more, in many cases much more, in a reliable, quantitative manner and without significant sacrifice in performance. To maximize impact, we have focused on making our add-on easy to assemble from only a few off-the-shelf components, and to implement with no requirement of microscope hardware modifications.

## Materials and Methods

### Active illumination

A layout of our system is presented in Supplementary Information. We use a Red Pitaya development kit featuring a Xilinx Zynq 7010 system on chip (CPU & FPGA) and integrated 125 MS/s 14 bit ADCs (2×) and DACs (2×). The fluorescence signal *S* obtained from the microscope PMT (Hamamatsu H7422PA-40 with Prairie Ultima pre-amp) is normally routed to the microscope acquisition electronics. Instead, we route it here to one of the ADC input of the Red Pitaya board, through a variable voltage divider (5 kΩ) adjusted so that the overall ADC gain *G*_*S*_ is nominally adjusted to fill the converter bit depth (minus one). That is, *G*_*S*_ = ${2}^{{B}_{S}}/{S}_{sat}$, where *B*_*S*_ = 13 and *S*_*sat*_ is the saturation signal obtained from the PMT. The error difference between the digitized signal $\hat{S}={G}_{S}S$ and a programmed set point ${\hat{S}}_{0}$ is then fed into a PID controller to compute the targeted digitized laser power $\hat{P}$ (see Supplementary Information for details^18^). This is sent to the EOM (Conoptics 350-80LA with 302RM driver) via one of the Red Pitaya DAC outputs such that the nominal overall DAC gain is *G*_*P*_ = *P*_*max*_/${2}^{{B}_{P}}$, where *B*_*P*_ = 14 and *P*_*max*_ is the EOM driver voltage corresponding to the maximum allowed laser power. This is adjusted using a variable voltage diver (5 kΩ) between the Red Pitaya output and the EOM driver input (alternatively, it can be loaded into the Red Pitaya FPGA through its webpage interface).

In the event that the target $\hat{P}$ is less than the maximum allowed ${\hat{P}}_{max}$ the board is in feedback-active mode. Otherwise the board automatically switches to power-limited mode and $\hat{P}$ is held fixed at ${\hat{P}}_{max}$. To turn active illumination off we bypass the feedback and apply a user-defined constant voltage to EOM.

### Log-encoding and reconstruction of sample strength

Regardless of whether the board is in feedback-active or power-limited mode, the sample strength *X* is computed from a knowledge of $\hat{S}$ (see above) and $\hat{P}$ (obtained by probing the laser power with a $\sim 2$% beamsplitter, neutral density filter, and a Thorlabs PDA36A amplified photodiode, and adjusted with a variable voltage divider to match the photodiode output range to the 1 V input range of the second Red Pitaya ADC input). Once computed and encoded by a logarithm operation, *X* is routed to the microscope acquisition electronics via the second Red Pitaya DAC output. For the logarithm operation, both $\hat{P}$ and $\hat{S}$ are converted to ${\mathrm{log}}_{2}\hat{P}$ and ${\mathrm{log}}_{2}\hat{S}$ using a lookup table containing *B*_*L*_-bit entries pre-calculated by MATLAB and stored in onboard ROM. These entries are scaled by *G*_*L*_ = ${2}^{{B}_{L}}$/*B*_*S*_, and processed to obtain ${\hat{L}}_{X}\equiv {G}_{L}{\mathrm{log}}_{2}\hat{X}+C={G}_{L}({\mathrm{log}}_{2}\hat{S}-\alpha {\mathrm{log}}_{2}\hat{P})+C$, where *C* is an additive constant chosen to avoid the possibility of negativities in ${\hat{L}}_{X}$ in the event the microscope acquisition electronics does not allow negative input levels (we used here *B*_*L*_ = 13 and *C* = ${2}^{{B}_{L}}$). After ${\hat{L}}_{X}$ is output by our active illumination unit D/A converter it is then re-digitized by the microscope acquisition electronics. The link between ${\hat{\hat{L}}}_{X}$ after re-digitization and ${\hat{L}}_{X}$ is given by ${\hat{\hat{L}}}_{X}={G}_{M}{\hat{L}}_{X}$, where ideally *G*_*M*_ is adjusted to fill the bit depth *B* of the microscope acquisition electronics when ${\hat{L}}_{X}$ is maximum. What is displayed on the computer screen, which, as far as the microscope is concerned corresponds to a standard image, is then ${\hat{\hat{L}}}_{X}$. If desired, the numerical reconstruction of *X*, to within an unimportant scaling factor, can be performed post-hoc using the simple relation $X\propto {2}^{({\hat{\hat{L}}}_{X}/{G}_{L}{G}_{M})}$. Note that an accurate knowledge of *G*_*M*_ is critical here to ensure that the reconstruction of *X* is properly linear.

### Code availability

The Red Pitaya software and operation manual for the particular active illumination implementation described here will be made freely available on our web site: http://biomicroscopy.bu.edu.

### Mouse brain imaging

All samples were imaged with a Prairie Ultima two-photon microscope using an Olympus 20× NA 1.0 objective. Structural imaging of fluorescently labeled cortical pyramidal neurons was performed *in vitro* in acute, 300 *μ*m thick brain slices prepared from YFP-M mice using standard techniques^1^. Functional Ca^2+^ imaging was performed *in vivo* in the olfactory bulb of transgenic mice with widespread expression of the genetically encoded indicator GCaMP3^2^. Animals were anesthetized with isoflurane, and a craniotomy was performed over the dorsal olfactory bulb. Sensory responses to odorants were measured in mitral cells at a depth of 300–350 *μ*m from the dorsal surface. Odorants were amyl acetate and 2-methyl butyraldehyde, diluted in mineral oil to a concentration of 2%, and added to the main airstream using a custom olfactometer for a final concentration of 1%. All animal procedures were approved by the Boston University Institutional Animal Care and Use Committee and carried out in accordance with NIH standards.

## Electronic supplementary material

SI video 1

SI video 2

SI video 3

SI video 4

SI video 5

SI video 6

SI video 7

SI video 8

SI video 9

Supplementary Information
