# Supplementary material for: Neuronal imaging with ultrahigh dynamic range multiphoton microscopy
Source: Sci Rep. 2017 Jul 19;7:5817. doi: 10.1038/s41598-017-06065-7 (PMC5517475; doi:10.1038/s41598-017-06065-7)
Supplement: Supplementary file 10 — Supplementary Information [file 41598_2017_6065_MOESM10_ESM.pdf]

Neuronal imaging with ultrahigh dynamic range multiphoton microscopy: Supplementary information

Ruohui Yang[1], Timothy D. Weber[1], Ellen D. Witkowski[2], Ian G. Davison[2], and Jerome Mertz[1]

[1]Boston University, Dept. of Biomedical Engineering, 44 Cummington Mall, Boston, MA, 02215
[2]Boston University, Dept. of Biology, 5 Cummington Mall, Boston, MA, 02215

ABSTRACT

We provide a theoretical analysis of active illumination, and present a brief description of the algorithm and design of our FPGA-based device.

Theoretical calculation of SNR and dynamic range gain

Our goal here is to evaluate the expected gain in dynamic range provided by active illumination. For this, we will evaluate the expected SNR of our system.

We begin by writing $S = XP^{\alpha}$, where S is the fluorescence signal in units of number of detected photons per sample time (i.e. unitless), X is the sample strength, P is the illumination power, and α is the multiphoton order. We also define:

S_0 = set point for signal level when active illumination is engaged.
S_{sat} = signal level at which detector saturates.
P_0 = laser power used for standard microscopy (w/o active illumination).
$P_{\max}$ = maximum laser power allowed when active illumination is engaged.
$\eta_S = S_0/S_{sat}$ = normalized set point for active illumination.
$\eta_P = P_0/P_{\max}$ = normalized laser power w/o active illumination.
$X_{sat} = S_{sat}/P_0^{\alpha}$ = maximum sample strength that can be measured w/o active illumination.
B = digitization bit depth for microscope acquisition electronics.
B_F = digitization bit depth for FPGA input.

Our measurement of X contains noise. That is, we can write $X = \langle X \rangle + \delta X$, where $\langle ... \rangle$ corresponds to an ensemble average and δX is the noise associated with any given measurement of X. The variance of this noise is denoted by $\sigma_X^2 = \langle \delta X^2 \rangle$, leading to a SNR associated with the measurement of X given by SNR = $\langle X \rangle / \sigma_X$. This definition of SNR will be used throughout.

Let us first calculate the SNR associated with standard multiphoton microscopy (i.e. without active illumination). In this case, the digitized signal can be written as $\hat{S} = \langle \hat{S} \rangle + \delta \hat{S}$, with

$$\langle \hat{S} \rangle = G_M \langle S \rangle$$

$$\langle \delta \hat{S}^2 \rangle = \langle G_M^2 \delta S^2 + \delta N_S^2 \rangle = G_M^2 \langle S \rangle + \sigma_{NS}^2$$

where G_M is the gain associated with the microscope acquisition electronics. The first term on the r.h.s. of $\langle \delta \hat{S}^2 \rangle$ corresponds to shot noise. To fill the bit depth of these electronics, we use $G_M = 2^B/S_{sat}$. Also σ_{NS}^2 is the variance of electronic noise associated with the detection of $\hat{S}$. This includes both detector noise (in post-digitized units) and inaccuracies introduced by the digitization process itself.

Since our goal is to measure X, we should in principle also have a knowledge of the laser power P_0. Let us assume a best case scenario where P_0 is known perfectly. We have then

$$X = \frac{S}{P_0^\alpha} = \frac{1}{P_0^\alpha}\frac{\hat{S}}{G_M},$$

and to first order

$$\frac{\sigma_X^2}{\langle X\rangle^2} = \frac{1}{\langle S\rangle} + \left(\frac{1}{G_M\langle S\rangle}\right)^2 \sigma_{NS}^2.$$

This may be rewritten in terms of X_{sat} and S_{sat}, obtaining:
Standard microscopy mode:

$$\frac{\sigma_X^2}{\langle X\rangle^2} = \left(\frac{1}{S_{sat}}\frac{X_{sat}}{\langle X\rangle}\right) + \left(\frac{1}{G_M S_{sat}}\frac{X_{sat}}{\langle X\rangle}\right)^2 \sigma_{NS}^2, \tag{1}$$

from which we readily derive the SNR associated with a measurement of X.

We proceed in a similar manner in the case of active illumination. We write $\hat{S} = G_S S$ and $\hat{P} = G_P P$, and $\hat{L}_X = \log_2 \hat{X} + C$, where C is a constant introduced to ensure $\hat{L}_X$ does not become negative. These quantities are digitized values processed by the FPGA board. The FPGA output $\hat{L}_X$ is then re-digitized when it is read by the microscope acquisition electronics, leading to $\hat{\hat{L}}_X = G_X \hat{L}_X + \delta N_X$, where δN_X takes into account inaccuracies due to digitization (i.e $\sigma_{N_X}^2 = 0.083$ for randomly distributed measurements). What is displayed on the computer screen is then $\hat{\hat{L}}_X$.

To evaluate the SNR associated with this measurement, we must numerically reconstruct X. This is given by

$$X = 2\hat{}\left(\frac{1}{G_X}\hat{\hat{L}}_X - C\right),$$

leading to

$$\langle X\rangle + \delta X = \frac{\left(\langle\hat{S}\rangle + \delta\hat{S}\right)}{\left(\langle\hat{P}\rangle + \delta\hat{P}\right)^\alpha} 2^{\delta N/G_X} \approx \frac{1}{\langle\hat{P}\rangle^\alpha}\left(\langle\hat{S}\rangle + \delta\hat{S}\right)\left(1 - \alpha\frac{\delta\hat{P}}{\langle\hat{P}\rangle}\right)\left(1 + \frac{\delta N_X}{G_X}\ln 2\right).$$

For ease of calculation, we introduce here an assumption. In the case when $\hat{P}$ is large, its measurement becomes shot-noise limited and $\delta\hat{P}/\langle\hat{P}\rangle \ll 1$ (i.e. its contribution can be neglected). In the opposite case when $\hat{P}$ is small, its measurement becomes electronic-noise limited, since it is detected with a simple photodiode. That is, we write $\langle\delta\hat{P}^2\rangle = \sigma_{NP}^2$. After some algebra, we obtain

$$\frac{\sigma_X^2}{\langle X\rangle^2} = \frac{1}{\langle S\rangle} + \left(\frac{1}{G_S\langle S\rangle}\right)^2 \sigma_{NS}^2 + \left(\alpha\frac{1}{G_P\langle P\rangle}\right)^2 \sigma_{NP}^2 + \left(\frac{1}{G_X}\right)^2 (\ln 2)^2 \sigma_{NX}^2.$$

Two scenarios must be considered since active illumination can operate in either feedback-active or power-limited modes. In feedback-active mode, we have $S = S_0 = \eta_S S_{sat}$ and $P^\alpha = S_0/X$, leading to

$$\frac{\sigma_X^2}{\langle X\rangle^2} = \frac{1}{\eta_S S_{sat}} + \left(\frac{1}{G_S\eta_S S_{sat}}\right)^2 \sigma_{NS}^2 + \left(\frac{\alpha}{G_P}\right)^2 \left(\frac{\langle X\rangle}{\eta_S S_{sat}}\right)^{2/\alpha} \sigma_{NP}^2 + \left(\frac{\ln 2}{G_X}\right)^2 \sigma_{NX}^2.$$

In power-limited mode, we have $P = P_{\max}$ and $S = P_{\max}^\alpha X = \frac{1}{\eta_P^\alpha} S_{sat}\frac{X}{X_{sat}}$, leading to

$$\frac{\sigma_X^2}{\langle X\rangle^2} = \frac{\eta_P^\alpha}{S_{sat}}\frac{X_{sat}}{\langle X\rangle} + \left(\frac{\eta_P^\alpha}{G_S S_{sat}}\frac{X_{sat}}{\langle X\rangle}\right)^2 \sigma_{NS}^2 + \left(\frac{\alpha}{G_P P_{\max}}\right)^2 \sigma_{NP}^2 + \left(\frac{\ln 2}{G_X}\right)^2 \sigma_{NX}^2.$$

In the event G_P is properly adjusted to fill the FPGA input bit depth, then $G_P = 2^{B_F}/P_{\max}$ and these expressions can be simplified further:

Feedback-active mode:

$$\frac{\sigma_X^2}{\langle X\rangle^2} = \frac{1}{\eta_S S_{sat}} + \left(\frac{1}{G_S\eta_S S_{sat}}\right)^2 \sigma_{NS}^2 + \left(\frac{\alpha}{\eta_P 2^{B_F}}\right)^2 \left(\frac{\langle X\rangle}{\eta_S X_{sat}}\right)^{2/\alpha} \sigma_{NP}^2 + \left(\frac{\ln 2}{G_X}\right)^2 \sigma_{NX}^2. \quad (2)$$

Power-limited mode:

$$\frac{\sigma_X^2}{\langle X\rangle^2} = \frac{\eta_P^\alpha}{S_{sat}}\frac{X_{sat}}{\langle X\rangle} + \left(\frac{\eta_P^\alpha}{G_S S_{sat}}\frac{X_{sat}}{\langle X\rangle}\right)^2 \sigma_{NS}^2 + \left(\frac{\alpha}{2^{B_F}}\right)^2 \sigma_{NP}^2 + \left(\frac{\ln 2}{G_X}\right)^2 \sigma_{NX}^2. \quad (3)$$

We note that the transition between feedback-active and power-limited modes occurs at a threshold sample strength given by $X_t = S_0/P_{\max}^\alpha = \eta_S \eta_P^\alpha X_{sat}$.

Let us now compare the maximum dynamic ranges of standard versus active illumination microscopes.

With standard microscopy, the minimum possible measure of X is $1/G_S P_0^\alpha$. The maximum possible measure is S_{sat}/P_0^α. The best dynamic range, corresponding to the ratio of the two, is then $G_S S_{sat}$.

With active illumination, the minimum possible measure of X is obtained in power-limited mode and given by $1/G_S P_{\max}^\alpha$. The maximum possible measure is obtained in feedback-active mode and given by $G_P^\alpha \eta_S S_{sat}$. Assuming G_P is properly adjusted, the best dynamic range is then $2^{\alpha B_F}\eta_S G_S S_{sat}$.

The potential dynamic range gain afforded by active illumination is thus $2^{\alpha B_F}\eta_S$.

A more conservative estimate of this dynamic range gain can be evaluated based instead on the rolloff in the SNR that occurs in feedback-active mode when X becomes large (see Fig. 1c). This rolloff occurs when uncertainties due to electronic or digitization noise in the measurement of P become larger than uncertainties due to the shot noise in S_0, which, according to Eq. 2, occurs when

$$\left(\frac{\alpha}{\eta_P 2^{B_F}}\right)^2 \left(\frac{\langle X\rangle}{\eta_S X_{sat}}\right)^{2/\alpha} \sigma_{NP}^2 > \frac{1}{\eta_S S_{sat}},$$

leading to rolloff sample strength defined by $X_r = 2^{\alpha B_F + 1}\eta_S \xi$, where $\xi = \left(\frac{\eta_P}{\alpha\sigma_{NP}}\sqrt{\frac{1}{\eta_S S_{sat}}}\right)^\alpha$. A reduced estimate for the gain in dynamic range becomes then $2^{\alpha B_F}\eta_S\xi$. This reduced estimate is likely overly conservative since the SNR at X_r still remains high.

At this point, it is instructive to attach numbers to our calculations. Parameters needed to establish SNR as a function of normalized sample strength $\langle X\rangle/X_{sat}$ are η_S, η_P, G_S, G_P, and S_{sat}. As an example (c.f. Fig. 1c), we use $\eta_S = 0.5$ and $\eta_P = 1$, where the latter ensures that photodamage cannot increase. We also assume the electronic gains are adjusted such that $G_S = 2^{B_F}/S_{sat}$ and $G_P = 2^{B_F}/P_{\max}$ (bearing in mind that we could do slightly better with $G_S = 2^{B_F}/S_0$). Finally, we need to calculate S_{sat} in units of number of detected photons per sample time. A typical PMT sensitivity is 10^5A/W and a typical PMT output saturation level is 100μA. Hence PMT saturation typically occurs at roughly 1nW, or 2.5×10^9 photons/s. Assuming a sampling time of 1μs and a PMT quantum efficiency of order 10%, we arrive at $S_{sat} \approx 250$.

FPGA-based PID design

Our feedback system is designed based on the Red Pitaya development kit. Red Pitaya is a FPGA development kit with a dual core ARM Cortex chip on board and features two 125 MS/s analog inputs and two 125 MS/s analog output ports. The hardware parameters and functional layout are summarized below.

Table 1. Red Pitaya Hardware Description

Function	Component	Specification
A/D Converter	LTC2145	14 bit 125 MS/s
D/A Converter	AD9767	14 bit 125 MS/s
SoC	Xilinx Zynq 7010	

As is illustrated in Fig. S1, our FPGA-based PID controller is composed of five functional blocks.

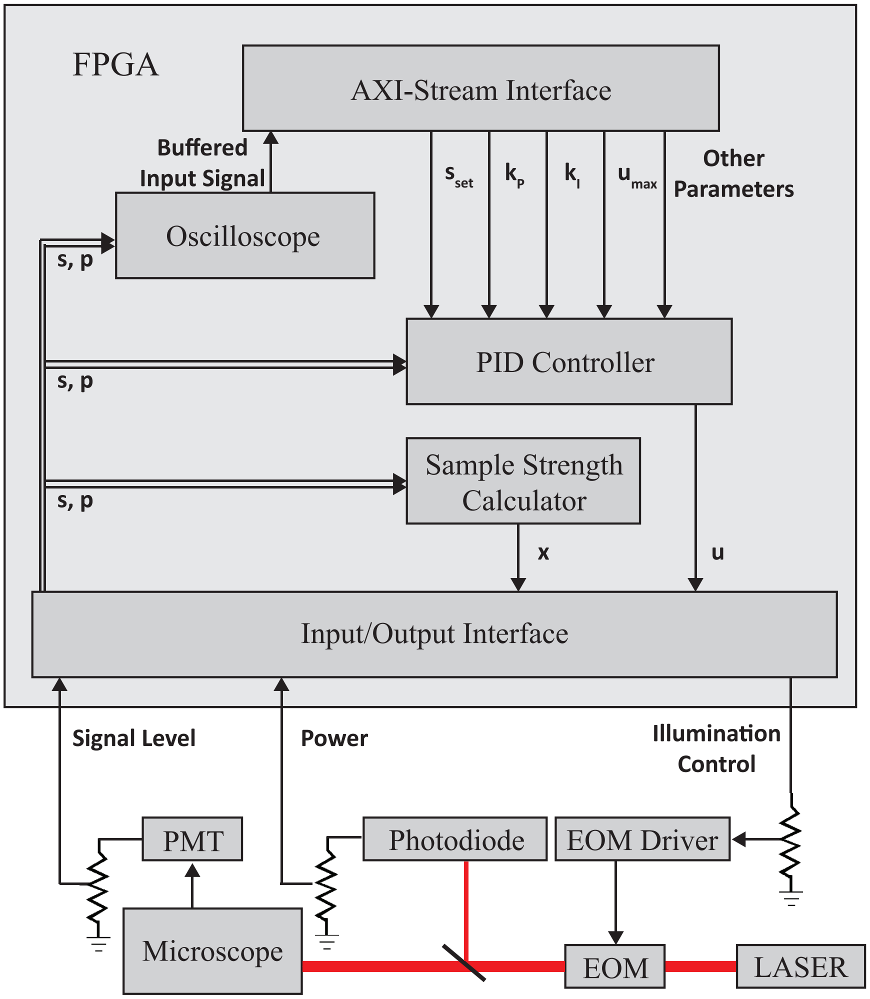

Figure S1: Overall structure of our PID controller. Functional blocks are shaded in gray. $s(k)$ is the discretized PMT signal; s_0 is the set point; k_P and k_I are feedback coefficients; u is the controller output signal, which controls the transmission of the EOM; p is the laser power, measured by the photodiode; x is the sample strength.

1. A PID controller that computes the desired controller output.
2. An AXI-Stream interface for the user to set the feedback parameters.
3. An oscilloscope to visualize the input signal.
4. A sample strength calculator.
5. An input/output interface.

Among them, the advanced extensible interface (AXI), the oscilloscope and the I/O interface is part of the Red Pitaya open source project. Detailed code and description can be found on github:

https://github.com/RedPitaya/RedPitaya/tree/master/fpga

The AXI-Stream interface provides real-time communication between Red Pitaya's Linux system and FPGA. We use it to adjust the feedback parameters and visualize the input signal. The input/output interface performs dual channel A/D and dual channel D/A conversion at 125 MS/s. In our case, signal level is digitized by input channel 1 while the illumination power is digitized by channel 2. The oscilloscope modules stores data from the high speed A/D converter in a triggered buffer and transfers the data through AXI-Stream to Red Pitaya's DRAM for on-screen visualization.

PID controller

Our PID algorithm is a widely-used negative feedback control algorithm that begins with a calculation of a time-varying error signal, $e(t)$, which is the difference between the values of the set point and current signal level. The error signal is sent to three contributing control parameters: a control directly proportional to the current error, a control based on the integrated past error signal, and a control based on the derivative of the error signal. The continuous PID control algorithm in standard form can be written as (12)

$$u(t) = K_p \left(e(t) + \frac{1}{T_i} \int_0^t e(t')dt' + T_d \frac{de(t)}{dt} \right). \tag{4}$$

In our case, we did not use the derivative component of the algorithm and set this to zero. As shown in Figure S2, we first subtract the input signal from the set point to obtain the error signal. We then multiply the error signal by a proportional gain k_p to obtain the proportional control term. At the same time, the error is multiplied by an integral gain k_I and added to a integral register. Finally, the proportional and integral terms are summed and the result is sent to an output actuator. The implementation of the algorithm contains two multipliers, two adders and one subtractor. To improve calculation speed, Xilinx®IP cores are used.

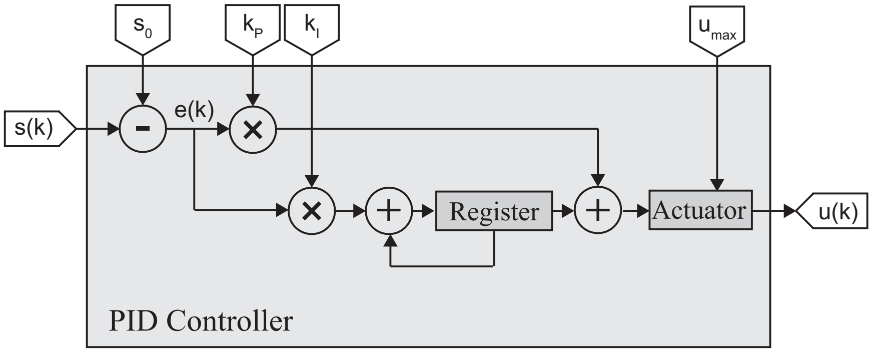

Figure S2: Work flow of the PID controller.

To alleviate the trade-off between feedback response time and ringing, we regulate the feedback gains in real time. In brief, when the imaging system scans through a dark area, the feedback gain is increased to reduce its response time; when the system scans through a bright area, the feedback gain is reduced to mitigate ringing.

Sample strength calculator

The use of a logarithm scale to encode sample strength was introduced in Chu *et al* (4). As shown in Figure S3, three steps are involved. First, the signal level is measured by the PMT and digitized as S and the illumination power is measured by the photodetector and digitized as P. Both S and P are encoded as 13-bit signals, meaning their logarithms can be computed using a same predefined and scaled lookup table that occupies less than 7KB of on-board ROM. Finally, a subtraction operation is performed between the logs of S and P (the latter being multiplied by 2 in the case of two-photon microscopy by bit shifting). A constant is added to the final result (here 2^{13}) before sending it to the microscope control system to prevent it from going negative.

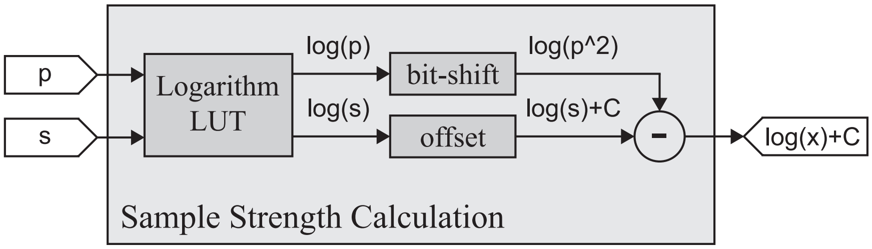

Figure S3: Work flow of the sample strength calculator.

FPGA Implementation

FPGA programming is performed through the Vivado®Design Suite 2015.1 by Xilinx. All source code is written in Verilog. The target FPGA device used in this research is the Xilinx Zynq 7010 SoC.

Device Resource Utilization

The device resource utilization is shown in Table 2.

Table 2. **Device Resource Utilization**

Resource	Utilization	Available	Utilization %
FF	4849	35200	13.90
LUT	4535	17600	25.77
I/O	257	6000	4.28
BRAM	33	60	55.00
DSP48	13	80	16.25
BUFG	7	32	21.88
PLL	1	2	50

Timing Analysis

There are several devices in our feedback loop, including a PMT, its associated transimpedance amplifier, and the EOM driver. Altogether, these devices were found to introduce a latency in the feedback of $\approx 2\mu$s. In contrast, the delays introduced by the FGPA operations equipped with a 125MHz oscillator as the master clock are:

1. A/D conversion. For the LTC2145 A/D converter, $\approx$ 7ns.
2. Digital signal processing, including signal wrangling, PID controller output computation and output thresholding, $\approx$ 10ns.
3. D/A conversion. For Red Pitaya's on-board D/A converter, $\approx$ 30ns.

The total latency of $\approx$ 50ns introduced by the FPGA is thus negligible compared to that introduced by the devices. In our case, the dominant speed limitation of our feedback comes from our EOM driver.

In our experiments we used a pixel sampling rate up to 1MHz (Figure S4 and Supplementary Video 9). Such a high pixel rate pushed our system to its very limit since barely a single control cycle is allowed per pixel. With our gain setting, the PID control can bring the PMT signal into a measurable range in the first cycle and thus avoid saturation. However, when only one cycle is allowed per pixel problems inevitably occur in PID feedback such as overshooting on upswings and undershooting on downswings. These problems are highlighted in Figs. S4 and S5, which illustrate active illumination at 1.2μs and 4μs pixel times. The sample strength $\log_2 X$, the raw PMT signal S, and illumination power P are simultaneously acquired with three channels of the microscope acquisition electronics. Ideally, the raw PMT signal should plateau to a constant value whenever the system toggles to feedback-active mode. Manifestly, even at 4μs pixel time, some overshooting occurs before the system is able to stabilize. Nevertheless, the calculated sample strength X remains unaffected because transient errors in S and P largely cancel one another.

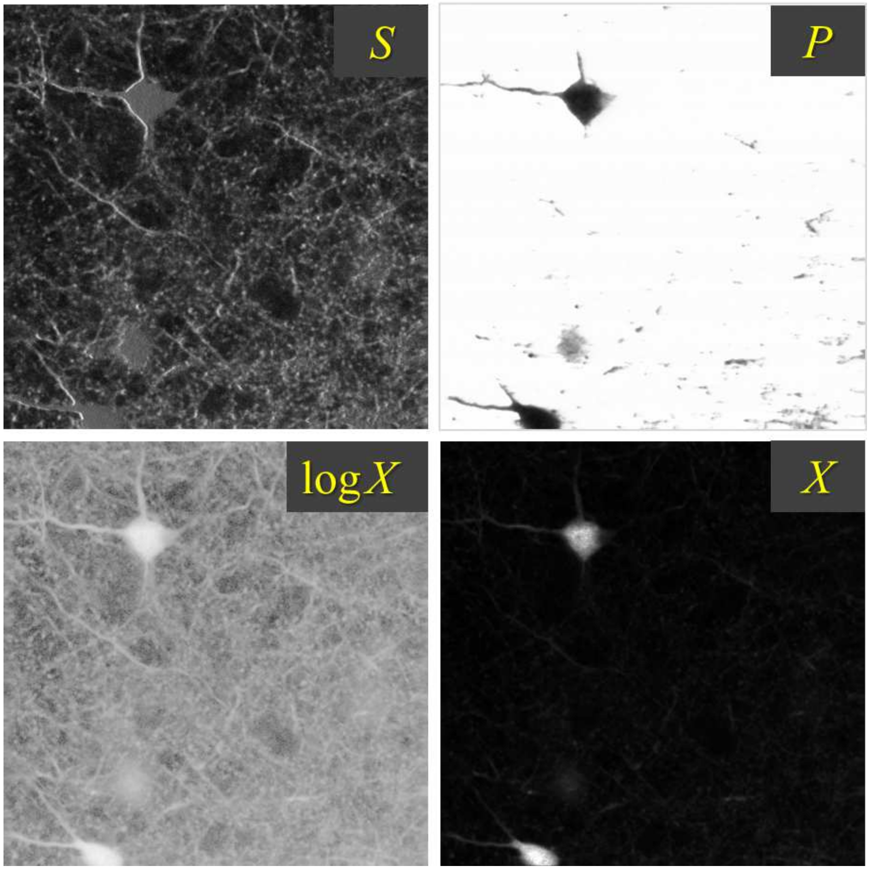

Figure S4: Images of YFP-labeled neurons in neocortical slice at 1.2μs pixel time (833kHz pixel rate). S is the raw PMT data while active illumination is on. P is the illumination power measured by the photodiode. $\log_2 X$ is the resultant log-encoded sample strength sent to the microscope acquisition electronics. X is the linear-encoded sample strength calculated post-hoc. Note the bright edges apparent in S are caused by overshoot in the feedback at 833kHz pixel rate. These are canceled after computation of $\log_2 X$ (or X).

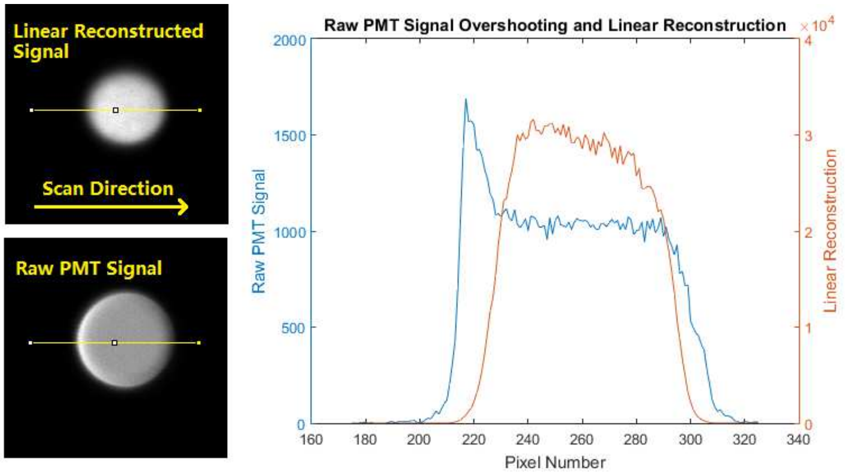

Figure S5: Image of a fluorescent bead. (left) Comparison of raw PMT signal S and reconstructed sample strength X while active illumination is on with 4μs pixel time. (right) Line plots (yellow line) are along the scan direction.

Reduced photobleaching with active illumination

Because AI effectively sets a limit on the fluorescence signal level when it is active, we expect it to also set a limit on the rate of photobleaching. To demonstrate this, we imaged a dendrite from a YFP-labeled cortical pyramidal cell. The total laser power incident on the slice was about 20 mW. We performed a sequence of 20 volumetric scans over the same region, where AI was ON for the first 10 scans and OFF for the remaining 10 scans. When AI was ON, no saturation occurred. When AI was off, occasional isolated spots in the dendrite caused saturation. We selected of region of interest (circumscribed in red) that avoided these spots. The total fluorescence signal produced from within this region is plotted as a function of time (or scan number). Manifestly, when AI is on, the photobleaching occurs at a slower rate than when AI is off, confirming the unsurprising result that the photobleaching rate becomes reduced with the reduced excitation power occasioned by AI.

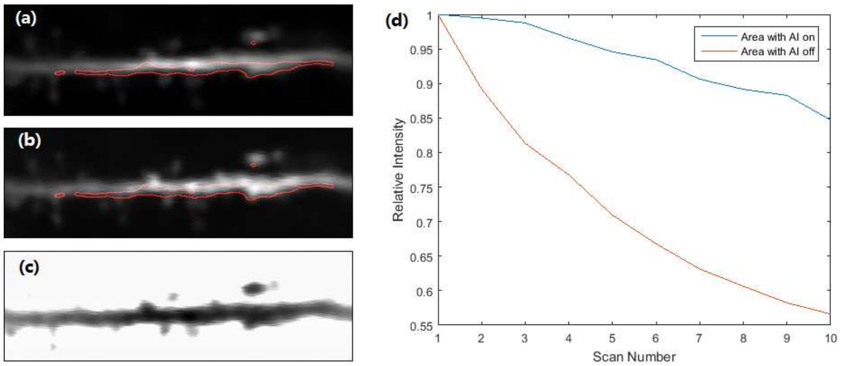

Figure S6: A YFP-labeled dendrite repeatedly imaged with (a) AI on and (b) AI off. The darker regions in (c) correspond to regions where AI became active. The total fluorescence power recorded from a region of interest (circumscribed in red) is plotted as a function of scan number. The plots are normalized to their respective starting levels. When AI is ON, the photobleaching rate is slower than when AI is OFF.

Supplementary Video 1

YFP-labeled pyramidal neuron in neocortical slice (26-frame 75μm z-stack, 3.2μs pixel time). Left: conventional 2-photon microscopy (AI off, linear scale) with sufficient illumination power to reveal dendritic structures, causing brighter cellular compartments to saturate detector. Right: active illumination (AI on, log scale) reveals weak dendritic structures while avoiding saturation. Both panels correspond to what is directly recorded by the microscope acquisition electronics.

Supplementary Video 2

YFP-labeled pyramidal neurons in neocortical slice (23-frame 88μm z-stack, 3.2μs pixel time). Left: conventional 2-photon microscopy (AI off, linear scale) with illumination power set to just avoid saturation at bright somata. Right: active illumination on (AI on, log scale) reveals weak dendritic structures while avoiding saturation. Both panels correspond to what is directly recorded by the microscope acquisition electronics.

Supplementary Video 3

Mitral/tufted cells and dendrites in the olfactory bulb of Thy1-GCaMP3 mice subjected to odor stimulation (3.2μs pixel time; 3.2 fps). Conventional 2-photon microscopy (AI off) with illumination power set for low probability of saturation.

Supplementary Video 4

Mitral/tufted cells and dendrites in the olfactory bulb of Thy1-GCaMP3 mice subjected to odor stimulation (3.2μs pixel time; 3.2 fps). Conventional 2-photon microscopy (AI off) with illumination power increased to improve SNR (at the cost of allowing saturation).

Supplementary Video 5

Mitral/tufted cells and dendrites in the olfactory bulb of Thy1-GCaMP3 mice subjected to odor stimulation (3.2μs pixel time; 3.2 fps). Reconstructed (i.e. linearized) video obtained from active illumination (AI on), exhibiting good SNR and no saturation.

Supplementary Video 6

Mitral/tufted cells and dendrites in the olfactory bulb of Thy1-GCaMP3 mice subjected to odor stimulation (3.2μs pixel time; 5.2 fps). Conventional 2-photon microscopy (AI off) with illumination power set for low probability of saturation (note: saturation occurs nonetheless).

Supplementary Video 7

Mitral/tufted cells and dendrites in the olfactory bulb of Thy1-GCaMP3 mice subjected to odor stimulation (3.2μs pixel time; 5.2 fps). Conventional 2-photon microscopy (AI off) with illumination power increased to improve SNR (at the cost of allowing saturation).

Supplementary Video 8

Mitral/tufted cells and dendrites in the olfactory bulb of Thy1-GCaMP3 mice subjected to odor stimulation (3.2μs pixel time; 3.2 fps). Reconstructed (i.e. linearized) video obtained from active illumination (AI on), exhibiting good SNR and no saturation.

Supplementary Video 9

YFP-labeled neurons in neocortical slice (48-frame 144μm z-stack, 1.2μs pixel time). Left: conventional 2-photon microscopy (AI off, linear scale) with illumination power set to just avoid saturation at bright somata. Right: active illumination (AI on, log scale). Both panels correspond to what is directly recorded by the microscope acquisition electronics.
